# Effect of Spectral Filtering and Segmental X-ray Tube Current Switch-Off on Interventionalist’s Scatter Exposure during CT Fluoroscopy

**DOI:** 10.3390/bioengineering11080838

**Published:** 2024-08-16

**Authors:** Oliver S. Grosser, Martin Volk, Marilena Georgiades, Daniel Punzet, Bahaa Alsawalhi, Dennis Kupitz, Jazan Omari, Heiko Wissel, Michael C. Kreissl, Georg Rose, Maciej Pech

**Affiliations:** 1 Department of Radiology and Nuclear Medicine, University Hospital Magdeburg and Medical Faculty of Otto-von-Guericke University, 39120 Magdeburg, Germany; martin.volk@med.ovgu.de (M.V.); marilena.georgiades@med.ovgu.de (M.G.); bahaa.alsawalhi@st.ovgu.de (B.A.); dennis.kupitz@med.ovgu.de (D.K.); maciej.pech@med.ovgu.de (M.P.); 2 Research Campus STIMULATE, Otto-von-Guericke University, 39106 Magdeburg, Germany; daniel.punzet@ovgu.de (D.P.);; 3 Chair of Medical Telematics and Medical Technics, Institute of Medical Engineering, Faculty of Electrical Engineering and Information Technology, Otto-von-Guericke University, 39106 Magdeburg, Germany

**Keywords:** CT fluoroscopy, ambient dose exposure rate, radiation protection, dose optimization

## Abstract

Dose optimization in computed tomography (CT) is crucial, especially in CT fluoroscopy (fluoro-CT) used for real-time navigation, affecting both patient and operator safety. This study evaluated the impact of spectral X-ray filtering using a tin filter (Sn filter), and a method called partial-angle computed tomography (PACT), which involves segmentally switching off the X-ray tube current at the ambient dose rate ${\dot{H}}^{*}(10)$ at the interventional radiologist’s (IR) position. Measurements were taken at two body regions (upper body: head/neck; lower body: lower legs/feet) using a 120 kV X-ray tube voltage, 3 × 5.0 mm CT collimation, 0.5 s rotation speed, and X-ray tube currents of 43 Eff.mAs (without Sn filter) and 165 Eff.mAs (with Sn filter). The study found significant dose reductions in both body regions when using the Sn filter and PACT together. For instance, in the upper body region, the combination protocol reduced ${\dot{H}}^{*}(10)$ from 11.8 µSv/s to 6.1 µSv/s (*p* < 0.0001) compared to the protocol without using these features. Around 8% of the reduction (about 0.5 µSv/s) is attributed to the Sn filter (*p* = 0.0005). This approach demonstrates that using the Sn filter along with PACT effectively minimizes radiation exposure for the IR, particularly protecting areas like the head/neck, which can only be insufficiently covered by (standard) radiation protection material.

## 1. Introduction

Image-guided navigation by applying computed tomography (CT) fluoroscopic imaging is an established procedure in interventional radiology [1]. Different methodological approaches have been implemented, e.g., based on multiple consecutive helical CT scans of the target volume during the interventional procedure or by using real-time CT fluoroscopy (fluoro-CT) aids during the intervention [2]. The known hazardous aspects of X-rays have led to the determination of the ICRP dose limits (e.g., for the eyes, this is strictly limited to 20 mSv/year [3]), and the nominal probability for detriment-adjusted cancer risk is 4.1–4.8% per 1000 mSv in adult workers [4]. Therefore, the intensive use of X-ray radiation in fluoro-CT applications requires sufficient optimization. For navigating with multiple helical CT scans, the focus of the optimization is on patient exposure [5]. In contrast, for interventional procedures based on fluoroscopic-CT guidance, optimization is focused on the exposure of the patient and the workers in the operating room (e.g., interventional radiologist [IR], technician, and anesthesiologist) [6,7]. In addition to general concerns regarding hazards from ionizing radiation, special attention should be paid to the exposure of radiosensitive organs (e.g., eye lenses) and the identification of possibilities for optimizing personal protective equipment and protective shields (e.g., ceiling-mounted shields) [6,8,9,10]. Different aspects were analyzed, e.g., typical hand exposure to IR from different procedures performed in co-planar interventional techniques or patient exposure in fluoroscopic-guided procedures. The reported results generally reflect the local technical setting defined by the specific equipment (e.g., available interventional software packages for specific CT scanners and specific hardware features) [6,7,8]. In this context, different technical and methodological developments—e.g., the use of iterative image reconstruction in fluoro-CT, the improvement of CT detector technology, and advanced fluoroscopic-CT scan protocols acquiring projection data over less than a full 360° rotation of the CT gantry (partial-angle CT [PACT]) scanning only a defined angular range, also known as angular beam modulation (ABM)—have demonstrated significant effects for dose optimization in interventional procedures [7,11,12,13,14,15].

In addition, new techniques are being developed to allow for further reduction in the exposure to CT fluoroscopy. A potentially beneficial technical application is the filtering of X-ray tube spectra using an additional tin filter (Sn filter). Therefore, a hardened energy spectrum was obtained for imaging [16]. This specific filter technology, evaluated for diagnostic applications, e.g., dual-energy CT imaging or ultra-low-dose CT protocols, primarily absorbs low-energy photons that contribute negligibly to CT image quality but increase the radiation dose burden for the patient significantly [17,18,19,20,21]. However, the impact of this methodological development on the modulation (minimization) of the ambient dose exposure of the IR by scattered X-ray radiation has not been studied yet.

Owing to the known physical effects of the backscattering of photons (e.g., reduced backscattering for high-energy photons) [22], a reduction in the IR exposure level from backscattered photons may be achieved using the Sn filter technique.

In this study, the effect of the additional filtering of the X-ray spectrum (Sn filtering) on the IR exposure rate of scattered X-ray photons was examined. To determine the setting with the lowest exposure rate from scattered photons during an interventional procedure, the Sn filter technique was combined with the segmental PACT current, and the total effect on scatter exposure of the IR was evaluated. Examined CT scan protocols represent the manufacturer’s recommendation for image-guided procedures using fluoro-CT. For comparison, the corresponding CT exposure levels were reported for the protocols.

We present the general setting for fluoro-CT scanning used in our study (Section 2.1), the experiments performed for measuring the ambient dose exposure rate from scattered X-ray photons using Sn filtering and combinations of Sn filtering with PACT in fluoro-CT (Section 2.2), the expected patient exposure resulting from the protocol being investigated (Section 2.3), and the statistical methodology used for data evaluation (Section 2.4). The results are described and discussed in Section 3 and Section 4, respectively.

## 2. Materials and Methods

### 2.1. Two-Dimensional Fluoro-CT

**Figure 1:**
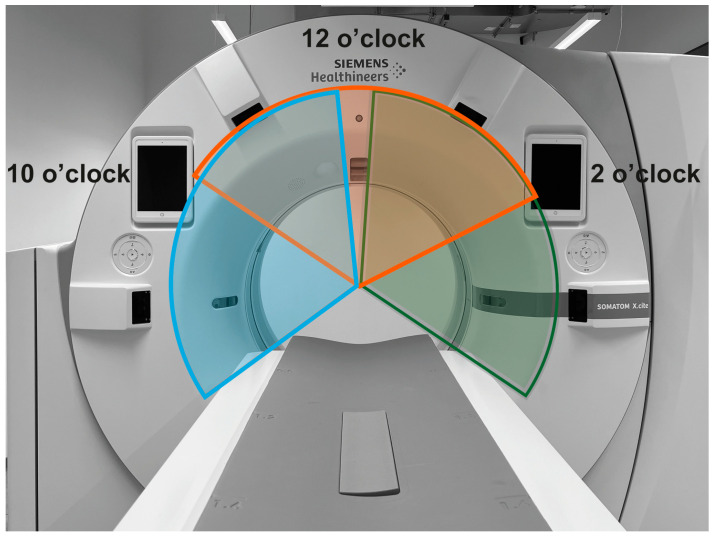
Angular segments with X-ray tube current switched off (blue: 10 o’clock segment, orange: 12 o’clock segment, and green: 2 o’clock segment).

The study was performed using a clinically available CT scanner (Somatom X.cite, Siemens Healthineers, Erlangen, Germany). The CT scanner affords different scan modes for CT-guided interventions: (1) 3D-CT data from helical scanning, (2) step-and-shot scanning, and (3) 2D-fluoro-CT imaging. In this study, dose exposure from fluoro-CT imaging was examined. The scanner was equipped with a hardware feature for the optional filtering of the X-ray spectrum using a Sn filter in an X-ray tube collimator box [16]. In addition, the CT scanner features PACT data acquisition using fluoro-CT protocols (HandCARE™ option, Siemens Healthineers, Erlangen, Germany). As a result, fluoro-CT imaging was performed using a PACT scan under optionally strictly limited angular scan conditions. The segmental off-switching of the X-ray tube current can be used in three different settings (Table 1 and Figure 1) [23].

Spectral filtering using the Sn filter and the PACT scan methodology can be used separately or in combination. Furthermore, the effects of Sn filtering without the influence of a system feature restricting the emissions of the X-ray tube to the defined angular segments were examined. In this setting, the starting point for the scanned angular segment was defined for each scan based on the actual position of the continuously rotating X-ray tube.

The examined fluoro-CT protocol featured an X-ray tube voltage of 120 kV, a primary CT collimation of 15.0 mm (slicing 3 × 5.0 mm), and a rotation speed trot of 0.5 s. Scans were performed with an effective X-ray tube current corresponding to 43 Eff.mAs in the standard setting (without additional filtering) and with 165 Eff.mAs while using the additional Sn filter [17]. Protocol parametrization was performed according to the manufacturer’s recommendations for 2D-fluoro-CT. The scan protocols were optimized by the manufacturer to provide a comparable CT exposure level for both Eff.mAs settings [11].

The additional modulation of the X-ray tube current (e.g., angular modulation depending on patient diameter) comparable to diagnostic CT protocols is not available for the fluoro-CT scan mode.

### 2.2. Exposure to Scattered Radiation

**Figure 2:**
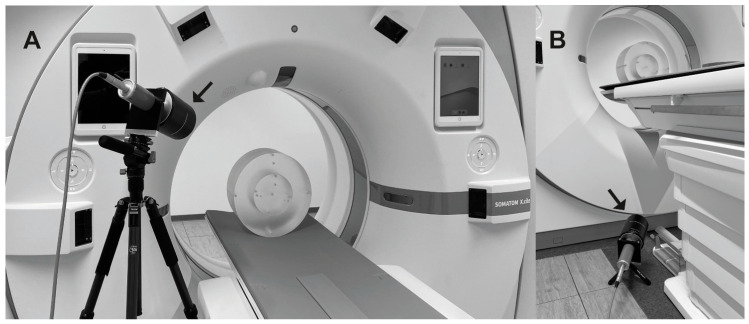
Examined setup with CTDI-Phantom positioned centrally in the CT gantry with detector chamber positioned (**A**) in the height of IR upper body and (**B**) in the position of the lower legs/feed. The respective position of the detector is indicated by an arrow.

The ambient dose exposure ${\dot{H}}^{*}(10)$ from scattered X-ray photons was measured using a calibrated survey meter (model: Survey Meter OD-02, STEP—Sensor Technic and Electronic, Pockau, Germany) with a large ionization chamber (chamber volume, V = 600 cc). The dose rate meter provided a homogenous sensitivity profile in 180° geometry and was approved for the measurement of pulsed radiation. For each fluoro-CT scan, the shortest possible scan duration (X-ray tube current on, t_scan_ = 0.34 s) was selected. The ambient dose rate ${\dot{H}}^{*}(10)$ was calculated accordingly. Scatter radiation was generated by scanning a standardized cylindrical polymethylmethacrylate (PMMA) phantom position in the isocenter of the CT (CTDI-Phantom, PTW, Freiburg, Germany) with a length of 15 cm, which is usually used in CTDI_vol_ measurements [24]. The phantom featured a diameter of 32 cm and was representative of the (abdominal) body geometry of a normal patient [25]. Dose measurements were performed beside the right side of the gantry (right-hand side of a patient in the supine position head-first to the CT gantry, approximately 30 cm in front of the gantry) for two positions typically representing the region of the upper body (including head/neck; dosimeter chamber height 130 cm above floor level) and lower body region (lower legs/feet; dosimeter chamber height 15 cm above floor level) of the IR (Figure 2A,B).

For each combination of position and scan protocol setting (e.g., with/without off-switching of the X-ray tube current at specific angular positions in combination with/without a Sn filter), the measurements were repeated 10 times.

### 2.3. Patient Exposure

For examined PACT scan protocols, with/without using the Sn filter in combination with the other scan protocol-specific setting (X-ray tube current and voltage, collimation, etc.), the Computed Tomography Dose Index [CTDI_vol_] and dose-length product [DLP] reported by the CT system were documented as surrogates for a patient’s CT exposure.

### 2.4. Statistics

The R software package (version 4.13; R Foundation for Statistical Computing, Vienna, Austria) was used for statistical evaluation. Descriptive parameters were expressed as means ± standard deviations or medians, interquartile ranges (IQR), and ranges, if appropriate. All data were tested for normality using the Kolmogorov–Smirnov test. General effects from factors, e.g., from the examined body region, the usage of a Sn filter, or a combination of parameters, on ${\dot{H}}^{*}(10)$ were determined via generalized linear model analysis. Pairwise comparisons of ${\dot{H}}^{*}(10)$ for the identification of significant differences between different factor levels (e.g., segmental X-ray tube current switching, with/without a Sn filter) were performed by using the Wilcoxon rank-sum test with Bonferroni–Holm correction applied for multiple comparisons. All tests were two-sided, and significance was assumed at *p* < 0.05.

## 3. Results

### 3.1. Effect of Using a Sn Filter

**Figure 3:**
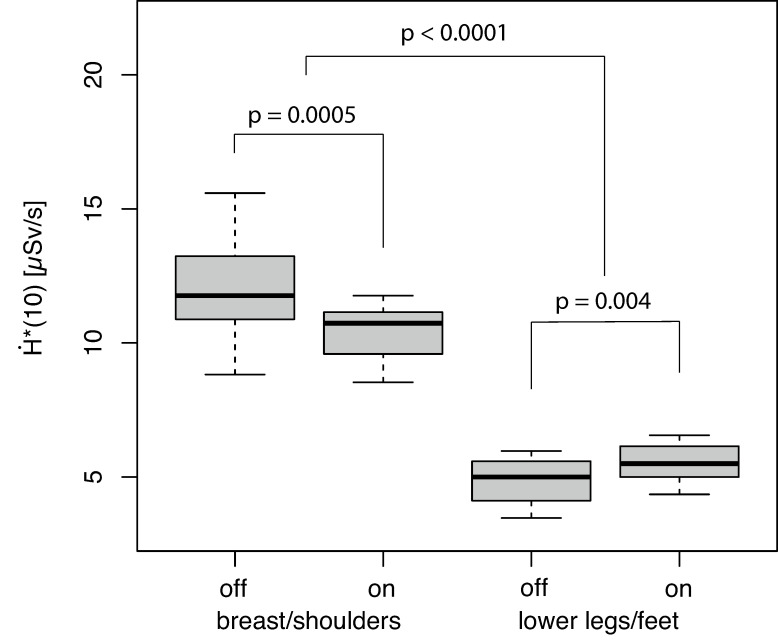
Effect from additional spectral filtration (off = without Sn filter, on = with Sn filter) on ${\dot{H}}^{*}\left(10\right)$ in the examined body regions.

The effect of the Sn filter was evaluated by measuring ${\dot{H}}^{*}(10)$ for the standard fluoro-CT protocol (segmental off-switching of the X-ray tube current was not activated). The ${\dot{H}}^{*}(10)$ values were not normally distributed (*p* ≤ 0.0001). A significant effect of spectral filtration using a Sn filter on ${\dot{H}}^{*}(10)$ was observed in both of the examined body regions (*p* ≤ 0.004, Figure 3).

The dose rate significantly decreased in the upper body region using a Sn filter by a median of 12.5% (absolute 1.49 µSv/s). In contrast, the dose rate increased upon using the additional filter in the region of the lower legs/feet by a median of 12.7% (absolute 0.62 µSv/s) in comparison to the measurements without additional filtering. The dose rate in the region of the upper body was approximately 1.8–2.5 times higher relative to the lower legs/feet region (Figure 3, *p* < 0.0001).

### 3.2. Effect of Combining Sn Filter with PACT

The ${\dot{H}}^{*}(10)$ values observed for the scan conditions (segmental switching and Sn filter) were not normally distributed (*p* ≤ 0.0001). Significant effects from the examined body region, the usage of a Sn filter, PACT parametrization (e.g., the position of the segmental off-switching of X-ray tube current), and the combination of both parameters on ${\dot{H}}^{*}(10)$ were determined via generalized linear model analysis (each effect, *p* ≤ 0.0001).

**Figure 4:**
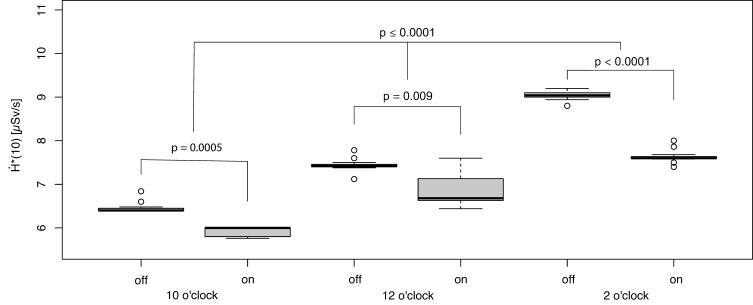
Effect from spectral filtration (off = without Sn filter, on = with Sn filter) in combination with the segmental X-ray tube switching off on ${\dot{H}}^{*}\left(10\right)$ in the upper body.

In the upper body region, the lowest ${\dot{H}}^{*}(10)$ was observed for the fluoro-CT protocol with an active Sn filter and activated segmental off-switching of the X-ray tube current in the 10-o’clock position (Figure 4).

The reduction owing to using the Sn filter was by a median of 8.1% (absolute 0.53 µSv/s) in comparison to the identical PACT setting without the additional Sn filtering of the X-ray spectrum. The reduction in ${\dot{H}}^{*}(10)$ by PACT in combination with the Sn filter was compared to the fluoro-CT protocol without PACT and a Sn filter by a median of 48.9% (*p* < 0.0001, reduction by a median of 11.85 µSv/s to 6.06 µSv/s; Figure 3 and Figure 4). The other examined fluoro-CT settings (PACT settings) generally provided significantly higher exposure rate levels (Figure 4); however, additional spectrum filtering always resulted in an individual reduction in ${\dot{H}}^{*}(10)$ (*p* ≤ 0.0009).

**Figure 5:**
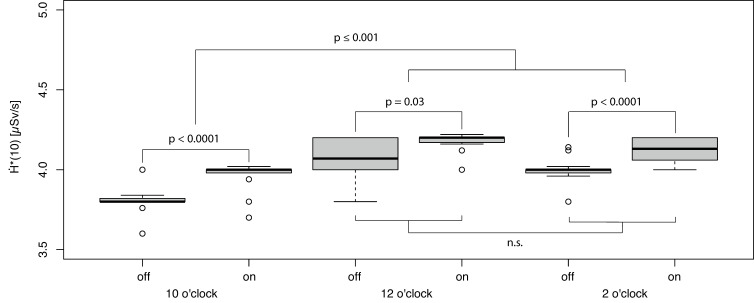
Effect from spectral filtration (off = without Sn filter, on = with Sn filter) in combination with the segmental X-ray tube switching off on ${\dot{H}}^{*}(10)$ in the region of the lower legs and feet (n.s.—not significant).

In contrast, the lowest dose rate in the region of the lower leg/feet was observed for the fluoro-CT setting without using the Sn filter in combination with X-ray off-switching at 10 o’clock (Figure 5).

In the corresponding setting with the Sn filter, representing the optimum in the upper body region, the exposure rate increased by 4.3% (absolute 0.17 µSv/s). Additionally, all other combinations of spectral filtering (with/without a Sn filter) in combination with further X-ray switching configurations (e.g., PACT with 12 o’clock and 2 o’clock settings) showed an increased dose rate relative to the fluoro-CT setting without using the Sn filter and the off-switching of the X-ray tube current in the 10 o’clock position.

### 3.3. Patients’ CT Exposure

From the patients’ perspective, the individual scan setting defined by variations in pre-filtering (Sn filter) and PACT parametrization was correlated with a comparable CT exposure level documented by CTDI_vol_ (DLP) measures. In the setting without an additional Sn filter (X-ray tube current corresponding to 43 Eff.mAs) and with a Sn filter (X-ray tube current corresponding to 165 Eff.mAs), a CTDI_vol_ of 4.06–4.09 mGy/s (DLP: 6.09–6.13 mGy×cm/s) and 4.13–4.16 mGy/s (DLP: 6.20–6.24 mGy×cm/s) was observed, respectively. The Sn filter-based scan protocol increased CT exposure by 1.98% (ΔCTDI_vol_ = 0.08 mGy/s, *p* < 0.0001). The exposure correlates with a single fluoro-CT scan (shortest possible scan).

## 4. Discussion

In the current study, spectral filtering using an additional Sn filter, a technology established for diagnostic CT imaging, was investigated regarding the potential of IR exposure optimization in fluoro-CT. In addition, the combination of this technique with the established methodology of PACT on the IR exposure rate was examined. The primary objective was to identify the fluoro-CT protocol with the lowest CT IR exposure rate. We focused on two representative body regions: (1) the upper body regions (including the head and neck) and (2) the lower legs/feet. The exposure level was analyzed for body regions of the IR that are usually not directly or not optimally covered by radiation protection equipment (e.g., due to procedure-specific patient access). To our knowledge, the study represented the first evaluation of the effect of spectral pre-filtering on IR exposure rate by scattered photons. The examined imaging protocols represent the manufacturers’ recommended fluoro-CT procedures.

In using an additional Sn filter, we observed a significant reduction in ${\dot{H}}^{*}\left(10\right)$ in the upper body region of the IR in direct contrast to the identical protocols without the corresponding filtering. In contrast, exposure in the region of the lower legs/feet was increased using the Sn filter. However, as expected, the dose exposure was lower than that in the upper body region owing to the larger distance to the scattering medium. In both scenarios, we discuss energy-dependent effects in the Compton scattering of X-ray photons, generally derived from Klein–Nishina formalism [22]. The exposure rate in the upper body region can be addressed by the known shift in the effectively observed energy emission to higher energies by the Sn filter [16]. In this context, the backscattering probability is decreased compared with the standard setting without Sn filtering. In contrast, the exposure level in the lower body region increased when using the Sn filter. This can be discussed by effects in the forward scatter probability. The forward scatter probability of photons increases with an increase in the primary photon energy, and the effect of the increased primary photon energy on the angular distribution of forward-scattered photons must be considered (e.g., the lateral component projecting to the position of the IR becomes more pronounced) [26]. In conjunction with the different aspects, the observed changes in the exposure rate of scattered X-ray photons on IRs can be addressed. Combining the segmental switching of the X-ray tube current with the Sn filter methodology provides further opportunities for optimizing the dose exposure in the upper body region. The lowest exposure rate of the upper body region was identified for the scan protocol, switching the X-ray tube current off in the segment directly corresponding to the IR side. In parallel, the observed increase in the exposure rate in the lower body region (lower legs/feet) underlines the requirement for an optimized radiation protection setting covering X-ray photons below the CT table [27]. However, the lower body region can be easily shielded (e.g., by lead curtains). Despite this limitation, this combination has a significant advantage. The ambient exposure rate in the working field of the IR, requiring good and sterile access to the patient (always limited by the gantry diameter, in conjunction with the patient, and limited spatial access) with limited capabilities for additional radiation protection equipment (e.g., concerns regarding collision and sterility) was significantly reduced. Using a different interventional strategy (e.g., patient access from the opposite side of the gantry) requires an adapted parametrization of the PACT. Following the optimization strategy of the manufacturer, the CT exposure (CTDI_vol_, DLP) was for both Sn settings in a comparable dimension.

In general, the phantom geometry, which was used as a scattering medium in our study, must be discussed from a methodological point of view due to its geometry deviating from the patient’s geometry. Several authors reported comparable scattering properties for the CTDI phantom and the anthropomorphic phantoms [28,29,30]. The geometry used can, therefore, be regarded as suitable and can be easily reproduced for validation and for further optimization in fluoro-CT-imaging (e.g., analyzing effects on image quality by spectral filtering) due to the good availability of these phantoms.

Meanwhile, the comparison of absolute exposure rates is hampered by the different positions and dimensions of the scatter geometries. In contrast to diagnostic CT applications, patients are often positioned off-center for fluoro-CT-guided interventions (e.g., at a lower table position or laterally shifted). Modifications in patient positioning were performed to improve patient access inside the CT gantry for handling interventional equipment (e.g., needles for biopsy or applicators for radiofrequency or microwave ablation). Consequently, different exposure scenarios have been observed for phantom setups by other authors [14,31]. Direct comparison is further affected by the different configurations of the examined imaging systems (e.g., system-specific X-ray spectrum, primary collimation for fluoro-CT imaging), the phantom geometry used to generate the scattered photons, and the methodology used for reporting the exposure level (effective dose, ${\dot{H}}^{*}(10)$). In this context, the CT covers (including the system components behind them, e.g., the material of the CT gantry) and the patient table must be considered as a further source of scattered photons. Additionally, the measurement of the scatter is generally ambiguous, with the risk of underestimating ambient exposure (or exposure rate) using dosimeters with different (and limited) angular sensitivities, small detector volumes, inappropriate spectral characteristics, or the inability to perform measurements in a pulsed and rotating scatter field.

Furthermore, the analysis of eye lens exposure is of interest in a specific field of application. Although direct measurements were not possible with the equipment used, the effects of ${\dot{H}}^{*}(10)$ still illustrate the potential for minimizing eye lens exposure using these technologies.

Generally, the optimization of radiation protection settings is indisputable because of the effects of radiation (early aging, carcinogen effects, cataracts, cognitive impairment, etc.) [4]. In parallel, IRs treat an increasing number of patients with more complex interventions using new sophisticated devices. This situation was further accentuated by the limited number of IRs. Training plays an important role in interventional radiology, as it raises professional qualifications [32,33,34]. In this context, the need for the further evaluation and optimization of radiation protection in interventional procedures is an inherent element for the further development of interventional radiological procedures. Further possibilities for dose optimization can be discussed for hybrid techniques combining different imaging modalities (e.g., using ultrasound for navigation in conjunction with fluoro-CT to reduce the CT scan time) [35]. In our study, the specific effects of the Sn filter in combination with/without PACT, e.g., effects on image quality owing to the use of an X-ray spectrum hardened by the Sn filter in comparison to image quality generally known through standard fluoro-CT, were not examined. Examinations of image quality by applying fluoro-CT are always limited by numbers and missing generalized quality standards [7]. The focus is on fluoro-CT protocols provided by the manufacturer, which generally represent the starting point for any optimization. In addition, when using PACT, the timely resolution is limited to two reconstructed fluoro-CT images per second. In contrast, the fluoro-CT protocol without angular constraints enables a rate of 8–10 reconstructed images per second. However, the effect of limited timely resolution must be questioned in fluoro-CT-guided interventions.

## 5. Conclusions

Finally, knowledge of the in-room ambient dose rate profile and the effect of different system features for optimizing IR dose exposure (e.g., Sn filtering, segmental off-switching of the X-ray tube current) is essential for tailoring the system-specific radiation protection setup. In this setting, personal radiation protection equipment is one part of the integral concept, in addition to the further optimization of the specific features of the imaging device and training. In this context, the Sn filter in combination with the segmental switching off of the X-ray tube current provides a technical–methodological opportunity for reducing IR exposure by around 49% (around 8% of the reduction is attributed to the usage of a Sn filter). The observed increase in exposure in the lower region of the body can be compensated for by stationary shielding in the corresponding area (below the CT patient table). In general, the examined features reduce the exposure rate level by utilizing available technology and can be implemented as a standard in the corresponding scan protocol. 

## Figures and Tables

**Table 1:** Parameterization of the angular segments with X-ray tube current switched off in PACT scan protocols.

Protocol Name	Segments with X-ray Current Off ^1^
10 o’clock	300° (250–350°)
12 o’clock	0° (310–50°)
2 o’clock	60° (10–110°)

^1^ Note: values are the central angle of the segment, and the range is shown in parentheses.

## Data Availability

The dataset is available upon request from the authors.
